# Systolic dysfunction associated with carfilzomib use in patients with multiple myeloma

**DOI:** 10.1038/s41408-017-0026-7

**Published:** 2017-12-13

**Authors:** Tania Jain, Hemalatha Narayanasamy, Joseph Mikhael, Craig B. Reeder, P. Leif Bergsagel, Angela Mayo, A. Keith Stewart, Farouk Mookadam, Rafael Fonseca

**Affiliations:** 1 Division of Hematology and Medical Oncology, Mayo Clinic, Phoenix, AZ USA; 2 Division of Cardiology, Mayo Clinic, Phoenix, AZ USA

Proteasome inhibition with carfilzomib (CFZ) has shown to be effective therapy in multiple myeloma (MM), with a response rate of around 87% and improved overall survival, in combination with lenalidomide and dexamethasone in patients with relapsed and refractory disease^1, 2^. Notably cardiac failure have been noted in 6–8% patients in these phase 3 trials, with 3–5% being grade 3 or higher. In a pooled analysis of 4 phase 2 clinical trials, cardiac failure was reported in approximately 7% patients and resulted in treatment discontinuation in 1.5% patients^3^.

The exact mechanisms behind cardiac dysfunction with CFZ are unknown. Some have postulated disturbance of endothelial nitric oxide synthase and nitric oxide^4^. In a previous report from our group, the toxicity appeared to be the result of an endothelial effect given the co-occurrence with hypertension and elevation in creatinine^5^. While cardiac toxicity has been reported with bortezomib^6^, it appears to be more frequent with CFZ, possibly because CFZ is more potent and binds irreversibly to the β-subunit of the 20 S proteasome^7^. This potency may also unmask potential protein metabolism consequences of proteasomal inhibition to the myocardium, a muscle in perpetual motion. Thus far, the predisposition, natural history, and reversibility of cardiac dysfunction are not well known. We share our experience of the clinical course of 12 patients who had worsening of cardiac function after initiation of CFZ.

We identified 136 patients at Mayo Clinic in Arizona who received CFZ treatment for MM between July 2012 and June 2016. Twelve of those were known to develop worsening of cardiac function upon exposure to CFZ. A decrease in ejection fraction (EF), on transthoracic echocardiogram evaluation, by 5% resulting in EF < 55% with symptoms of congestive heart failure or drop in EF by 10% resulting in EF < 55% regardless of signs or symptoms, was defined as a *cardiac event or cardiac dysfunction* in this study. For patients who had a baseline EF less than 55%, any decline in EF was noted as the systolic dysfunction. We conducted a retrospective review of patients’ charts to obtain demographic and treatment-related information. Known risk factors for congestive heart failure such as gender, cigarette smoking, overweight, hypertension, history of cardiac disease, previous exposure to anthracyclines were evaluated^8^. The study was approved by Institutional Review Board at Mayo Clinic Arizona.

## Patient demographics and risk factors

Median age at the time of initiation of CFZ therapy was 62.5 years (range, 55–70 years). Six (50%) patients were females. Median body mass index was 27.65 (range, 21–40) with 4 (33%) patients being obese. Two (17%) patients had a history of coronary artery disease while 9 (75%) had hypertension, and were on medical treatment at the time of CFZ therapy initiation. Baseline echocardiogram results were available for 10 of these 12 patients and median EF was 55% (range, 45–67%). Two (17%) patients had suffered previous episodes of congestive heart failure but their EF had improved by the time of initiation of CFZ therapy. In both these patients, EF was 55% at the time CFZ was started. Four (33%) patients had received anthracyclines in the past, cumulative dose of 100 mg/m^2^ doxorubicin in two patients, 120 mg/m^2^ liposomal doxorubicin in 1 and 240 mg/m^2^ doxorubicin in one patient. Details of baseline risk factors for each patient are described in Table 1.

**Table 1:** Baseline cardiac risk factors

Baseline cardiac risk factors:	Case#1	Case#2	Case#3	Case#4	Case#5	Case#6	Case#7	Case#8	Case#9	Case#10	Case#11	Case#12
Age at CFZ use/ Gender	69/M	57/M	57/M	70/M	66/M	61/F	63/F	68/M	63/F	62/F	62/F	55/F
Obesity (BMI>30)	Yes	No	Overweight	Overweight	Overweight	No	Yes	Overweight	Yes	Yes	Overweight	No
H/o smoking	No	No	No	No	No	No	No	No	No	No	No	Yes
H/o radiation therapy to chest wall	No	No	No	No	No	Yes	No	No	No	No	No	Yes
H/o anthracycline use*	No	No	No	No	No	No	Yes	Yes	No	Yes	No	Yes
H/o CAD	No	No	Yes	No	No	No	No	Yes	No	No	No	No
H/o CHF	Yes	No	No	No	No	No	Yes	No	Yes	Yes	No	No
H/o HTN	No	No	Yes	Yes	Yes	No	Yes	Yes	Yes	Yes	Yes	Yes
Baseline EF	53	67	N.A.	65	61	55	45	65	51	47	N.A.	55

*CFZ* carfilzomib, *M* male, *F* female, *H/o* history of, *any dose of anthracycline (no one received more than cumulative dose of 400 mg/m^2^), *CAD* coronary artery disease, *CHF* congestive heart failure, *HTN* hypertension, *EF* ejection fraction, *N.A*. not available, *overweight*—BMI = 25–29

### Treatment details

All patients had received multiple lines of therapy prior to starting CFZ (median 4; range, 2–7). All patients had previously received immunomodulatory therapy and 10 (83%) patients had received bortezomib. One (8%) patient had received VD-PACE (bortezomib, dexamethasone, platinum agent, doxorubicin, cyclophosphamide, and etoposide) and 10 (83%) had autologous stem cell rescue with high-dose melphalan.

### CFZ therapy and systolic dysfunction

Median duration of therapy with CFZ prior to the onset of systolic dysfunction was 4 months (range, 0.5–20 months). Three (25%) episodes happened within 1 month of CFZ initiation. Of the nine patients with baseline hypertension, three were noted to have worsening of blood pressure control along with systolic dysfunction. No hypertension was recorded in the three patients without previously known hypertension. A median reduction of 22% was noted in the EF (range, 9–45%), resulting in a median EF of 35% (range, 10–51%). N terminal pro-brain type natriuretic peptide (NT-pro BNP) level was available in seven patients and was elevated in all of these with a median of 4684 pg/ml (range, 901–32,740 pg/ml). Five of the 12 (42%) patients discontinued CFZ for other reasons prior to development of systolic dysfunction; however, they were included in the study since cardiac dysfunction happened shortly after treatment discontinuation at a median of 2 months (range, 0.5–3 months). Of the remaining seven, CFZ was stopped upon detection of cardiac dysfunction in five (42%) patients. Two patients (17%) continued to remain on CFZ therapy due to known aggressive disease, after extensive discussions with the patient and cardio-oncology team. In these patients, systolic function improved by 24% and 13%, respectively, despite continuing CFZ therapy.

### Outcomes

**Fig. 1:**
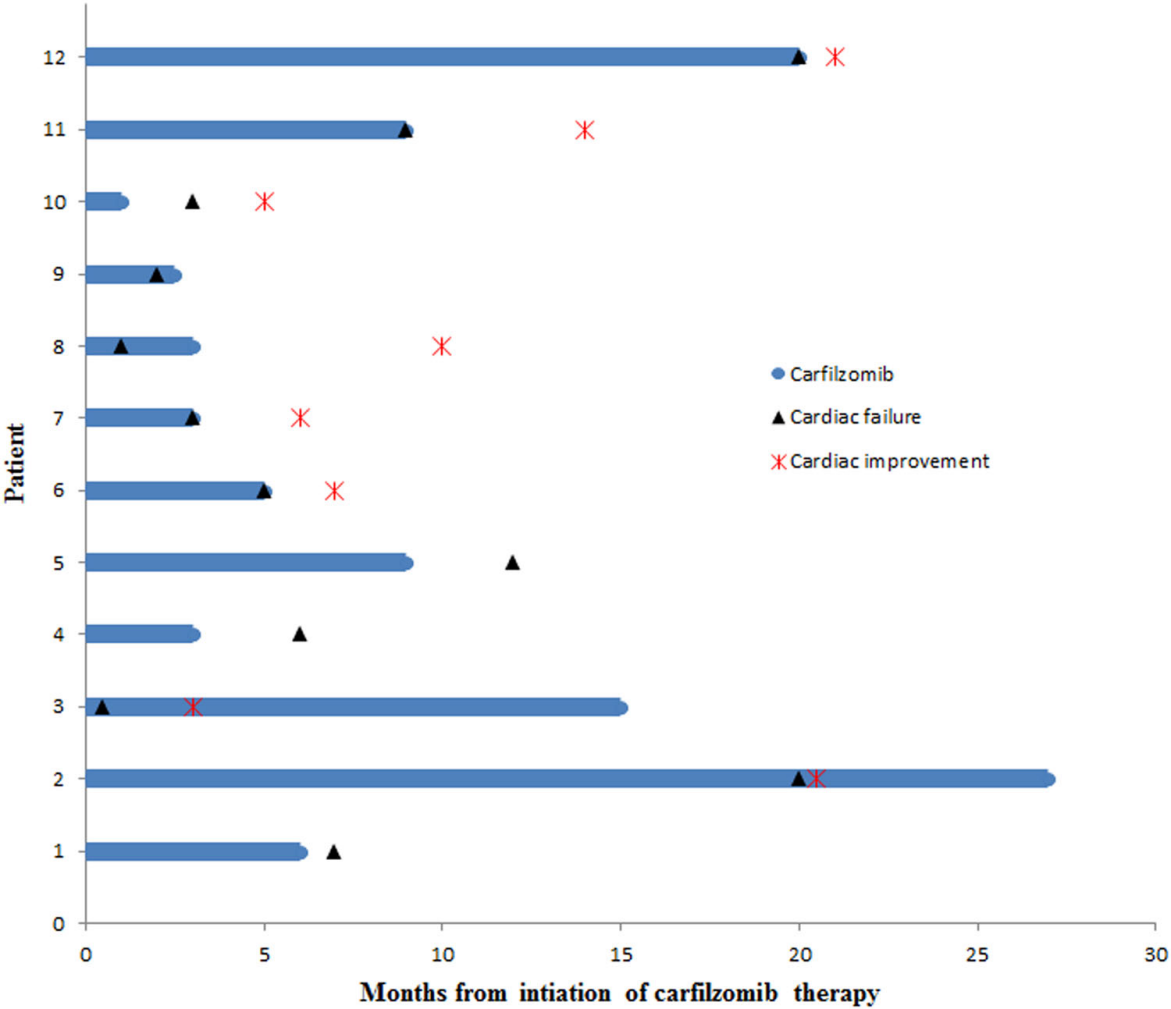
Timeline of events for all patients

Follow-up echocardiogram was available in 8 out of 12 patients. All of these patients showed improvement in EF. Median improvement in EF noted at the time of the last follow-up was 16% (range, 7–30%) at a median time of 2 months (range, 0.5–8 months). Median EF on follow-up echocardiograms was 48% (range, 35–67%). Although, repeat echocardiograms were not available on the remaining patients, their symptoms of dyspnea improved. One patient was re-challenged with CFZ, 8 months after initial cardiac event and discontinuation of the drug; and did not have recurrence of cardiac dysfunction. Timeline of events for each patient is depicted in Fig. 1.

No patients died from cardiac dysfunction secondary to CFZ use. Of these 12 patients, 6 (50%) had died by the time of last follow-up. The two patients, who continued CFZ, died of progressive MM and sepsis, respectively. Of the ten patients who discontinued CFZ, four (40%) had died from progressive MM in three and sepsis in one patient.

In this study, we sought to understand patterns of systolic dysfunction in patients who received CFZ. In our experience of 12 patients, it appears that all patients had an underlying risk factor for systolic dysfunction. This suggests that caution and careful management be used for patients ≥ 55 years of age, who have cardiovascular risk factors or who have an underlying cardiac dysfunction. Although no patients had received cumulative dose more than 350 mg/m^2^ doxorubicin (which is the dose associated with significant reduction in EF from baseline^9^), four patients had received a cumulative dose of 100–240 mg/m^2^ of doxorubicin. At these doses, the rates of congestive heart failure can range from 0.2–1.6% based on previous studies^10^. These rates are low and, hence, signify that even lower doses can contribute to subsequent cardiotoxicity. In another series, presence of underlying cardiovascular disease was associated with a higher incidence of cardiac events in patients undergoing treatment with CFZ.^11^ Not all patients with underlying cardiac dysfunction, however, develop systolic dysfunction from CFZ. Hence, it would be beneficial for clinical practice, to identify factors that can predict development of systolic dysfunction in these patients in larger studies. One prospective study showed that baseline EF, strain and *E*/*e*’ ratio were not predictive of cardiac toxicity from CFZ^5^.

Most patients in our study had systolic dysfunction early in the course of CFZ treatment. Data from previous trials also suggest that rate of cardiac adverse effects did not worsen beyond 12 cycles^4^. The magnitude of drop in EF was significant and led to symptoms of dyspnea in most patients. More importantly, systolic function was noted to improve in all patients on whom follow-up echocardiogram assessment was available. CFZ therapy was discontinued prior to or at the time of identification of cardiac dysfunction in most patients. However, there were two patients in whom CFZ therapy was never stopped and systolic function was still noted to improve shortly after the initial decrease. All patients were started on beta blocker, angiotensin convertase enzyme inhibitor and diuretic therapy. This data suggests that the systolic dysfunction can be improved with the use of appropriate therapy for systolic dysfunction and discontinuation of CFZ. Moreover, in all patients, the improvement was noted to occur promptly. This finding was similar to another case series of six patients in whom cardiac function was seen to recover after management with heart failure therapy and discontinuation of CFZ therapy^12^. This aspect is particularly important to recognize in patients who are being considered for therapy with CFZ, so as not to limit options in an incurable disease such as MM. Other cardiac toxicities reported in other single center studies include hypertension and arrhythmias, which should be monitored while on therapy^13^. Management strategies such as attention to fluid balance, reducing the rate of infusion, optimal management of hypertension should be considered to reduce cardiac events^14^.

Although small and limited by its retrospective nature, our study suggests caution or close monitoring in patients with pre-existing cardiac risk factors while undergoing CFZ therapy. Since echocardiograms were not regularly done at our center on patients who were receiving CFZ therapy, we cannot accurately assess the “incidence” of cardiac dysfunction from this study. Nevertheless, the systolic dysfunction improved in all patients. Ultimately, clinical assessment of risk vs. benefit of an individual case is necessary.
